# A Brunswick Stew

**Published:** 1893-11

**Authors:** C. A. Rominger

**Affiliations:** Reidsville, N. C.


ARTICLE X.

### A BRUNSWICK STEW.

BY DR. C. A. ROMINGER, REIDSVILLE, N. C.

Read before North Carolina State Dental Society, May, 1893.

### ANTRAL TROUBLES.

Diseases of the antrum, resulting from dental troubles, are by no means infrequent, and are often of a very grave character.

Mr. M., who had been treated by a physician for six months for catarrh, was suffering with engorgement of the antrum from an abscessed tooth. The floor of the orbit was so much elevated as to force the ball of the eye almost wholly out of the socket. On trial, the ball protruded an eighth of an inch beyond the ridge of the nose. The first superior molar on the left was extracted, as the offending cause, and an entrance made through the socket into the antrum, and then syringed out from the socket through the nose. It was so offensive that all windows and doors had to be opened to get rid of the awful stench.

Such cavities should first be thoroughly cleansed with peroxid of hydrogen, for several days, till the generation of pus ceases, and then followed by a 50 per cent. solution of campho-phenique in alcohol. This is antiseptic and, at the same time, is mild enough to stimulate the parts to healthy action.

Mrs. S. had suffered for sixteen years with chronic abscess of the antrum resulting from an offending molar. The tooth was removed shortly after the trouble began, but no entrance being made into the antrum and no drainage afforded, the disease smouldered on like the pent up fires of a volcano with occasional eruptions and emissions from the nose.

She was a great sufferer from what she and the physicians called neuralgia and catarrh. The bicuspids and first molar on the left were gone years before I saw the case. On close examination I found a little fold, or fissure, in the gums on the alveolar ridge near the position of the second bicuspid. The gums seemed to be healthy, but on forcing an explorer through the fissure, I found the bone to be in an unhealthy condition, not what you would call necrosed bone proper, but carious so that the probe would enter it and when pulled out it felt as if some clammy substance was cleaving to it.

The patient had suffered so long and so greatly that her mind was greatly impaired, and her constitution very much broken down. So without expressing a diagnosis at all, I cocainized the parts by injections, and bared the bone for an inch in length and half an inch in width along the alveolar ridge, and then with a large and sharp round bur I cut away all diseased bone. Then with a long implanting drill I made a free entrance into the antrum. Thus having free access into the antrum I washed out the cavity well discharging the medicine through the nose. This treatment was kept up for more than a week at intervals of two days each, and then the patient was dismissed with instructions for self treatment. She soon recovered the proper use of her mind and was restored to normal health.

POLYPUS.

Mrs. W. had a small growth attached to the superior maxilla just behind the left cuspid, which would bleed profusely on the slightest provocation. It gave no special pain, but had been a source of some anxiety. No diagnosis was given the patient; but the parts were well cocainized and the gum tissue cut away with a scalpel, and then the surface of the bone freely cut away with a bur at the point of attachment. The parts were well cleansed and antiseptisized, and the patient dismissed. All this was done at one sitting without ever alarming the patient with the fact that she had polypus and that it must be cut out. She was entirely cured.

Mrs. S. had a large gelatinoid polypus in each nostril, so as to completely close the air passages in damp weather. The attachment was so far up that I had to tear it away with an alligator forceps—some hemorrhage and considerable pain, so that the patient was dismissed for another time to extract the other. Not completed yet.

### TUMOR.

Mrs. M. had a tumor on the side of the nose just opposite the eye, about the size of a cherry seed. It had been coming for about seven weeks. The patient was very frail, and she and her husband was very nervous over it, fearing it was a cancer. With a hypodermic syringe I injected cocaine, and with little pain cut out a fatty tumor, to the great satisfaction and relief of the patient and her husband. I scraped out the cavity with a spoon shaped instrument, and then filled it with a piece of cotton saturated with campho-phenique, full strength. In about ten minutes I took that out, laid the edges of the wound together and covered with court-plaster. Dismissed.

### CATARRH.

Thousands of persons suffer with catarrh, who might be greatly relieved, if not permanently cured, by the intelligent dentist. Serious complications and unyielding sore throats often come from mouth-breathing; and mouth-breathing is most frequently the result of nasal stoppage. Frequently one nostril is nearly closed by spurs or prominences on the septurn of the nose, which can be removed by the skillful dentist; and the breathing passages nearly doubled by their removal, to the great relief and permanent benefit of the sufferer.

With a nasal speculum and a reflecting hand-mirror, the nasal passages can be explored with great satisfaction. Then by cocainizing the parts well, and with a good trephine on the dental engine the spurs can be almost painlessly removed. The wounded parts should be cleaned with peroxid of hydrogen, and the patient dismissed with the hope of a permanent cure. I have treated six cases recently of this character with the very best of results. It also greatly improves the hearing when it is impaired from catarrh of the eustachian tube.

### NEURALGIA.

I do not know of a more frequently used scape-goat than the one of the above name. I venture the assertion that neuralgia is not a disease *per se*. It is the manifestation of disease—the howling of the dog when the tail is mashed—the crying of the baby when the belly aches.

How foolish it would be to poultice the dog's mouth to cure the mashed tail, or to blister the baby's tongue to cure the belly ache! And yet many times when the immediate cause of a neuralgia is not at once found, by physician or dentist, the patient is dismissed with the consoling declaration that she has neuralgia, and thus she is abandoned to the agonies of purgatory without a ray of hope from those so-called lords of science. The most frequent cause of facial neuralgia is an exposed nerve or an alveolar abscess.

But severe facial neuralgias do frequently arise from other causes. One of the severest cases of facial neuralgia that ever came under my care was caused by albuminuria. I exhausted every resource I could command, and then called in the assistance of one of the most skilled physicians in the State, and we counselled together and searched diligently through the whole economy, at the same time trying every anodyne that we could summon for temporary relief, without the least particle of success. Finally we tested the urine and found it just freighted with albumin. A few days of treatment in cleansing the kidneys and stimulating them to healthy action, completely cured the neuralgia.

A lady came from Virginia to our town to be treated for sore throat and neuralgia in one side of the face, and while under treatment she came to my office for some work on her teeth. On examining them I found a lower molar on that side dead and having a large amalgam filling in it. It was not abscessed but only slightly tender to percussion. I said to her, "Well, now, I'll cure your sore throat;" and I did. I

put that tooth in good healthy condition, and the sore throat and neuralgia disappeared and never returned.

It is only the ignorant dentist or physician who treats neuralgia as a disease *per se*. Look for the cause and remove it, and the manifestation will at once disappear.

You may often need the assistance of an intelligent physician in such cases, especially if your patient is a female in her flowers, or during pregnancy. Ever keep a lookout for reflex pain, but look until you find the cause, and conscience as well as the gratitude of humanity will abundantly reward you for your labor.—*Southern Dental Journal.*

---

